# Type 1 diabetes and combined acute and chronic complications are associated with risk of progression of liver fibrosis: a Mendelian randomization study

**DOI:** 10.3389/fendo.2024.1302611

**Published:** 2024-08-05

**Authors:** Guangyuan Huo, Yueqiu Gao

**Affiliations:** Department of Hepatology, Shuguang Hospital Attached to Shanghai Chinese Medicine University, Shanghai, China

**Keywords:** type 1 diabetes, type 1 diabetes with acute complications, type 1 diabetes with chronic complications, risk of liver fibrosis progression, Mendelian randomization study

## Abstract

**Background:**

There has been controversy and uncertainty regarding the causal relationship between type 1 diabetes, its consequences, liver fibrosis, and cirrhosis. In order to determine the causal relationship, we conducted a Mendelian randomization study (MR).

**Methods:**

For the first time, we subjected multiple diabetes data to analyze its relationship with the progression of liver fibrosis. Once the instrumental variables had been extracted, we assessed them employing Cochran’s Q multi-analysis, inverse variance weighted, MR-Egger, MR-PRESSO, weighted mode, and weighted median.

**Results:**

Genetically predicted type 1 diabetes (OR = 1.13, 95% CI: 1.04–1.23, ^**^ *P* = 3.42 × 10^−3^), type 1 diabetes without complications (OR = 1.12, 95% CI: 1.03–1.23, ^*^ *P* = 1.26 × 10^−2^), type 1 diabetes with coma (OR = 1.09, 95% CI: 1–1.18, ^*^ *P* = 4.74 × 10^−2^), type 1 diabetes with ketoacidosis (OR = 1.07, 95% CI: 1.01–1.13, ^*^ *P* = 1.3 × 10^−2^), type 1 diabetes with neurological complications (OR = 1.18, 95% CI: 1.11–1.26, ^***^ *P* = 4.05 × 10^−7^), type 1 diabetes with ophthalmic complications (OR = 1.16, 95% CI: 1.05–1.28, ^**^ *P* = 3.06 × 10^−3^), type 1 diabetes with renal complications (OR = 1.07, 95% CI: 1–1.13, *P = 3.45 × 10^−2^), type 1 diabetes with other specified/multiple/unspecified complications (OR = 1.12, 95% CI: 1.02–1.23, ^*^ *P* = 1.41 × 10^−2^) were all associated with an increased risk of liver fibrosis progression.

**Conclusions:**

According to our MR investigation, type 1 diabetes and both its acute and chronic implications may increase the likelihood that liver fibrosis could continue to develop. Additionally, type 1 diabetes with neurological and ocular problems is more likely to accelerate the development of liver fibrosis and inflammation, which offers new insights for genetic investigations.

## 1 Introduction

A global health priority (1), type 1 diabetes (T1D) has witnessed a 3%–4% increase in prevalence over the past three decades (2). The risk of multiorgan complications, including acute complications [diabetic ketoacidosis, coma, etc. (3)] and chronic complications [ophthalmopathy, neurological, renal disease, etc. (4)], is increased by the imbalance in glycemic control triggered by insulin deficiency (5) brought on by pancreatic beta-cell autoimmunity. In recent years, we have witnessed an abundance of research on the liver, one of the organs related to type 1 diabetes (6).

A significant worldwide health issue is liver fibrosis induced by either viral or metabolic liver disease (7). Cirrhosis with structural loss, ensuing functional failure, and the emergence of life-threatening consequences have been defined as advanced chronic fibrosis (8). Liver fibrosis is a potentially reversible pathophysiological event (9, 10). Consequently, preventing the development of liver fibrosis to the point of cirrhosis is a potential way to prevent liver-related deaths (7). T2D boosts the risk of cirrhosis and liver-related mortality, particularly in people with NAFLD (11). Nevertheless, the link between liver fibrosis and the less prevalent type of diabetes, T1D, is rather uncommon and has generated debate. According to certain research, it results in little to no progression of liver fibrosis (12, 13), and according to two mate analysis studies, NAFLD patients with T1D experienced little to no progression of liver fibrosis (14, 15). According to certain observational studies, it may result in liver fibrosis (16, 17), which heightens liver stiffness (18, 19). One study revealed that 1 in 20 T1D patients had increased liver stiffness (12) or a 2.0% frequency of advanced fibrosis (15), whereas a Brazilian cross-sectional investigation indicated that 8.4% of T1D people had liver fibrosis (20). The most current MATE observational study (21) comprised just three investigations of T1D patients. However, virtually nothing has been established regarding the association between type 1 diabetes complications and the risk of liver fibrosis in the general population. Possible explanations include the low proportion of T1D patients recruited compared with T2D patients, the rarity of patients with comorbidities, and the length of certain studies, which makes it more challenging to perform research since patients are more likely to drop out. On the other hand, conclusions regarding the causal relationship between T1D, comorbid multisystem complications, and the degree of risk of liver fibrosis progression are hindered by the residual confounding of observational studies and the inherent difficulties of potential reverse causality, a challenge that may be overcome using MR methods.

Similar to RCT research, Mendelian randomization is an efficient statistical technique for determining causation based on whole-genome sequencing data (22). Public big data analysis is more effective and possesses a greater degree of proof as opposed to randomized controlled research, and it is capable of preventing bias and reverse causality. Previous MR research has investigated the risk factors for liver fibrosis and even cirrhosis, and MR studies have also discovered an association between the comorbidity of type 1 diabetes and NAFLD (23). However, neither of these two has previously been the subject of Mendelian randomization experiments.

In this study, we performed the first MR analysis of type 1 diabetes, its acute and chronic complications, liver fibrosis, and cirrhosis. Additionally, we investigated the possible causal relationship between these, as well as offered new suggestions for the early prevention and treatment of liver fibrosis progression and cirrhosis in patients with type 1 diabetes, especially in combination with acute and chronic complications.

## 2 Methods

### 2.1 Study design

**Figure 1:**
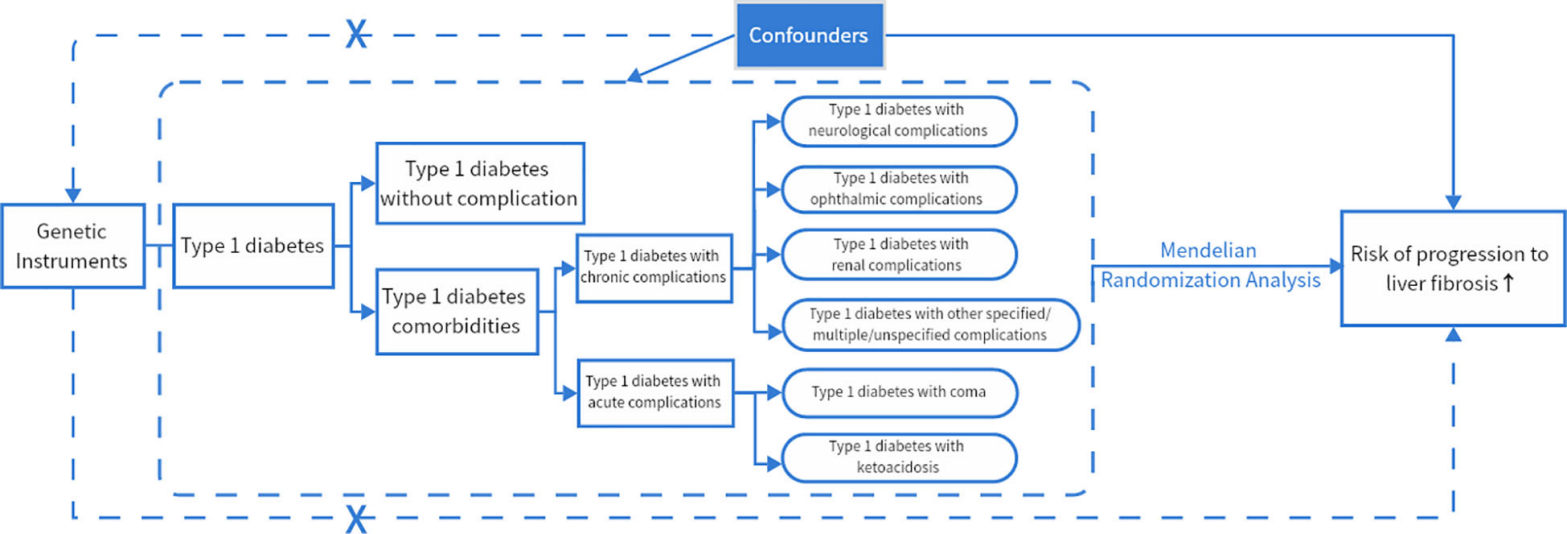
MR framework overview. If genetic variants (SNPs) can reliably control for disease risk factors (type 1 diabetes and its acute and chronic complications), and if there is a causal association between risk factors (type 1 diabetes and its acute and chronic complications) and disease endpoints (cirrhosis, liver fibrosis), then the genetic variants (SNPs) themselves should be associated with disease endpoints (cirrhosis, liver fibrosis). The assumptions required to produce unbiased estimates of causal effects as well as the rules of Mendelian randomization analysis of risk variables and risk of advancement of liver fibrosis can be modified. Potential pleiotropy or direct causality between variables is indicated by collapsed lines, which would go against the Mendelian randomization assumption.

This is a two-way two-sample MR and MVMR study, and an overview of the study design is presented in **Figure 1**.

### 2.2 Genetic epidemiology data sources

In the discovery phase, we utilized association statistics from databases and data from the FinnGen (https://www.finngen.fi/en/node/17) database based on gene-wide investigations. Type 1 diabetes (total), type 1 diabetes without complications, type 1 diabetes with coma, type 1 diabetes with ketoacidosis, type 1 diabetes with neurological complications, type 1 diabetes with ophthalmic complications, type 1 diabetes with renal complications, and type 1 diabetes with other specified/multiple/unspecified complications were among the variables selected for the genetic factor construction. The data were of European ancestry, and each cohort underwent GWAS, genotype estimation, and quality control pr (QC). At the same time, the initial GWAS was authorized by the appropriate institutional review board.

#### 2.2.1 Type 1 diabetes data sources

As the exposure factors, we specifically included type 1 diabetes (total) and type 1 diabetes without complications. Firstly, 188,103 European patients with type 1 diabetes without complications and 186,323 patients with type 1 diabetes had been selected based on phenotypic data as genetic instrumental variables for T1D. The enrolled patients were diagnosed with type 1 diabetes, both with and without complications, and the average age of first onset was 20.6 years. The absence of complications in type 1 diabetes diagnosis excluded complications associated with type 1 diabetes, diabetes caused by starvation, diabetes during pregnancy and delivery, neonatal diabetes, simple glycosuria, decreased glucose tolerance, and postoperative hypoinsulinemia. The mean age of first onset was 29.26 years.

#### 2.2.2 Type 1 diabetes with acute-complication data sources

As one of the exposure factors, we included type 1 diabetes with coma and type 1 diabetes with ketoacidosis. As genetic instrumental variables for type 1 diabetes paired with acute complications, 184,423 European patients with type 11 diabetes complicated by coma and 184,512 European patients with type 1 diabetes and ketoacidosis were identified. Type 1 diabetes-related comas with or without ketoacidosis, hypoglycemia coma, hyperosmolar coma, and hyperglycemic coma were all diagnosed. Patients experiencing both type 1 diabetic acidosis and type 1 diabetic ketoacidosis were diagnosed as type 1 diabetes with ketoacidosis.

#### 2.2.3 Type 1 diabetes with chronic complications data sources

The following complications were listed as one of the exposure factors: type 1 diabetes with neurological complications, type 1 diabetes with ophthalmic complications, type 1 diabetes with renal complications, and type 1 diabetes with other specified/multiple/unspecified complications. As genetic instrumental variables for chronic complications of T1D, 183,763 European patients with type 1 diabetes with neurological complications, 186,062 type 1 diabetes with ophthalmic complications, 184,148 type 1 diabetes with renal complications, and 186,601 type 1 diabetes with other specified/multiple/unspecified complications were selected. The diagnosis of type 1 diabetes with neurological complications includes type 1 diabetes with autonomic neuropathy, type 1 diabetes with mononeuropathy, type 1 diabetes with polyneuropathy, and type 1 diabetes with myasthenia gravis. The diagnosis of type 1 diabetes’ ocular complications includes type 1 diabetes with retinopathy and type 1 diabetes with cataracts. Type 1 diabetes with diabetic nephropathy, type 1 diabetes with intracapillary glomerulonephropathy, and type 1 diabetes with Kimmelstiel-Wilson syndrome were all diagnosed as having renal consequences. Type 1 diabetes with arthropathy, type 1 diabetes with neurologic diabetic arthropathy, type 1 diabetes with numerous complications, and type 1 diabetes with undefined complications were all diagnosed as type 1 diabetes with other specified/multiple/unspecified complications.

#### 2.2.4 Liver fibrosis and cirrhosis data sources

Cirrhosis and liver fibrosis were included as outcomes. The FinnGen database’s “K11_FIBROCHIRLIV” code was employed to select 214,403 European individuals with liver fibrosis and cirrhosis. The diagnosis excluded other liver disorders and included liver fibrosis, cirrhosis, liver fibrosis with cirrhosis, primary biliary cirrhosis, secondary biliary cirrhosis, biliary cirrhosis, and other undefined cirrhosis (e.g., cryptogenic, macronodular, mixed type). The mean age at the first onset of 63.88 years concentrated was between 50 and 80 years. The combined final report served as a genetic instrumental variable for cirrhosis and liver fibrosis. All of the above groups were not notably sex-specific.

### 2.3 Tool variable selection

To guarantee that there was an effect, three fundamental model assumptions of the MR analysis were satisfied. In order to prove that genetic variations (liver fibrosis, cirrhosis) and exposure (type 1 diabetes and its acute and chronic consequences) must be related, an association assumption was first created. The genetic variable *P* < 5e−08, and we expanded the study to set *P* < 5e−06 because there were too few genetic variables for type 1 diabetes with ketoacidosis, type 1 diabetes with neurological complications, and type 1 diabetes with renal complications. LD refers to non-random (nonrandom) associations between alleles (alleles) of different loci (loci) and is measured using two parameters r² and kb. We set the window to 10,000 kb and r² < 0.01 to ensure the independence of the selected genetic variants. Second, by employing the Phenoscan website, we eliminated confounding sociodemographic and disease state factors, such as age, obesity, heavy alcohol use, non-alcoholic fatty liver disease, hepatitis B or C infection, autoimmune disease, cholestatic disease, and iron or copper overload, which may contribute to liver fibrosis and cirrhosis. Finally, SNPs associated with outcomes were excluded and *P* ^2^ > 5 × 10^−5^ was set to satisfy the exclusivity assumption. In addition, F-value calculation was performed and SNPs with F <10 were excluded.

### 2.4 Statistical methods

#### 2.4.1 MR analysis method

For the two-sample MR analysis between exposure and outcome, the inverse variance weighted (IVW, random effects) method was employed. We first evaluated the causative association between liver fibrosis and cirrhosis and type 1 diabetes and its acute and chronic consequences using IVW as the major approach of analysis. It is a method for combining two or more random variables in order to reduce the aggregate variance, where the weight of each random variable in the sum is inversely proportional to its variance. Subsequently, as additional analysis approaches, we employed MR-Egger, weighted median (24), weighted mode, MR-PRESSO (25), and simple median. If the presumption that all contained SNPs may be utilized as genuine IVs is met, the IVW technique yields precise estimates (26).

#### 2.4.2 Sensitivity analysis to assess genetic markers

Employing MR-Egger, we failed to discover any evidence to support the null hypothesis of pleiotropy for genetic markers (*P*-value for pleiotropy > 0.05). We took into account the existence of an intercept term and utilized it to evaluate pleiotropy in the MR-Egger hypothesis. When the proportion of invalid instrumental variables is as high as 50% and the precision of the estimates varies substantially among instrumental variables, the weighted median method (27), defined as the median of the weighted empirical density function of the ratio estimates, continues to generate consistent effect estimates. Despite the fact that it is not as effective as IVW, the simple median nevertheless supports multiplicity (28). We utilized the “MR-PRESSO global test,” “MR-PRESSO outlier test,” and “MR-PRESSO distortion test” to eliminate anomalous SNPs (outliers) and estimate adjusted results. MR-PRESSO is a frequently employed method for testing horizontal multiplicity. To eliminate anomalous SNPs (outliers) and estimate the corrected results, researchers employed the “PRESSO outlier test” and the “MR-PRESSO distortion test”. The absence of horizontal pleiotropy effects for both variables was our final consideration. A prevalent test for heterogeneity is the Cochran Q statistic (29). The Cochran Q statistic is a standardized weighted sum of squares of the variances across studies, with smaller *P* values (usually at the level of α <0.10), indicating the presence of heterogeneity. the Q statistic usually exhibits a high statistical power when the number of included studies is large. Ultimately, we considered the absence of heterogeneity effects.

R (version 5.0.26) was utilized to analyze all of the aforementioned statistical analyses. The weighted median method, weighted mode method, and IVW method are all implemented in the TwoSampleMR R package. The MR-Egger analysis is performed by the MendelianRandomizationR program. The MR-PRESSO analysis is carried out through the MR-PRESSO R package.

## 3 Results

**Figure 2:**
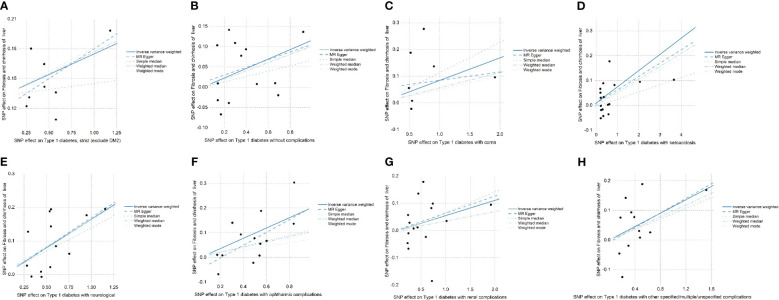
**(A–H)** The effects of SNPs on type 1 diabetes, type 1 diabetes with complications, liver fibrosis, and cirrhosis are displayed in a scatter plot. **(A)** Strict type 1 diabetes in patients with liver fibrosis and cirrhosis (exclude DM2). **(B)** Type 1 diabetes without complications in patients with liver fibrosis and cirrhosis. **(C)** Type 1 diabetes in patients with coma. **(D)** Type 1 diabetes in patients with ketoacidosis. **(E)** Type 1 diabetes in patients with neurological complications. **(F)** Type 1 diabetes in patients with ophthalmic complications. **(G)** Type 1 diabetes. **(H)** Type 1 diabetes with other specified/multiple/unspecified complications in liver fibrosis and cirrhosis. Mendelian randomization is referred to as MR and inverse variance weighted is referred to as IVW. The outcomes of these regression studies are represented by the IVW, MR-Egger, weighted median, weighted mode, and simple median slopes.

**Figure 3:**
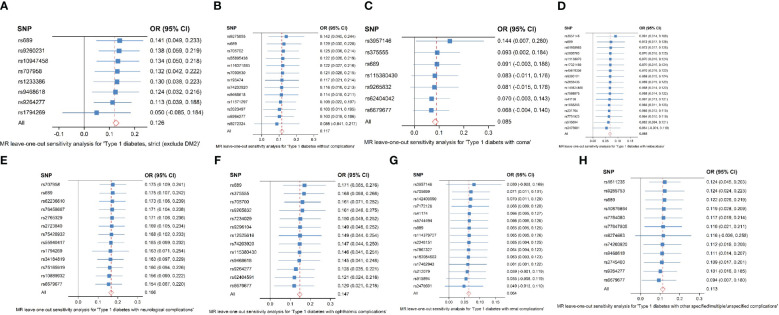
**(A–H)** Analysis of the “leave-one-out” causal association between type 1 diabetes and its consequences, including liver fibrosis and cirrhosis. **(A)** Strict type 1 diabetes in patients with liver fibrosis and cirrhosis (exclude DM2). **(B)** Type 1 diabetes without complications in patients with liver fibrosis and cirrhosis. **(C)** Type 1 diabetes in patients with coma. **(D)** Type 1 diabetes in patients with ketoacidosis. **(E)** Type 1 diabetes in patients with neurological complications. **(F)** Type 1 diabetes in patients with ophthalmic complications. **(G)** Type 1 diabetes with renal complications in liver fibrosis and cirrhosis. **(H)** Type 1 diabetes with other specified/multiple/unspecified complications in liver fibrosis and cirrhosis. The causal estimate and 95% confidence interval from the MR analysis after each SNP was removed are represented by the black dots and bars in the leave-one-out forest plot. The total MR estimate and 95% CI obtained using fixed-effect IVW approaches are represented by the red dots and bars.

SNPs with large outliers and those that departed from the symmetric midline utilizing scatter plots and funnel plots were initially omitted since they might be confounding factors in liver fibrosis and cirrhosis. We obtained eight separate sets of IVs without linkage disequilibrium (**Supplementary Material**). The bias of weak IVs was eliminated by the F statistics of these eight groups of IVs, which were all more than 1,000. Finally, for these eight groups, we created scatter plots (**Figure 2**) and leave-one-out plots (**Figure 3**).

**Figure 4:**
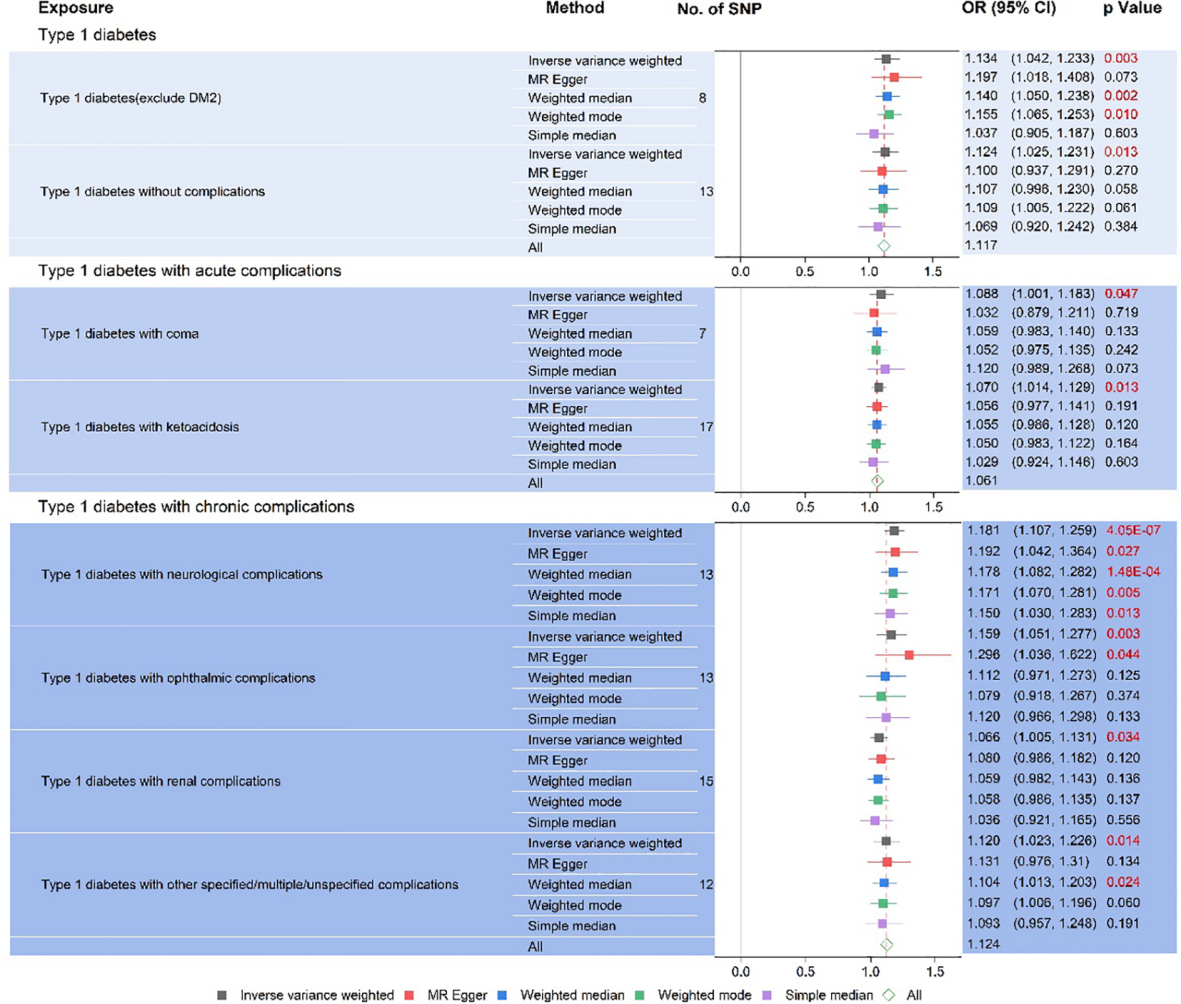
Type 1 diabetes and type 1 diabetes with acute and chronic complications on liver fibrosis and cirrhosis forest chart.

The results of the forest plot (**Figure 4**) presented a positive causal relationship between both type 1 diabetes and its complications and liver fibrosis and cirrhosis. Firstly, we performed IVW analysis, type 1 diabetes (exclude DM2): OR = 1.13, 95% CI: 1.04–1.23, ^**^ *P* = 3.42 × 10^−3^. Type 1 diabetes without complications: OR = 1.12, 95% CI: 1.03–1.23, ^*^ *P* = 1.26 × 10^−2^. Type 1 diabetes with coma: OR = 1.09, 95% CI: 1–1.18, ^*^ *P* = 4.74 × 10^−2^. Type 1 diabetes with ketoacidosis: OR = 1.07, 95% CI: 1.01–1.13, ^*^ *P* = 1.3 × 10^−2^. Type 1 diabetes with neurological complications: OR = 1.18, 95% CI: 1.11–1.26, ^***^ *P* = 4.05 × 10^−7^. Type 1 diabetes with ophthalmic complications: OR = 1.16, 95% CI: 1.05–1.28, ^**^ *P* = 3.06 × 10^−3^. Type 1 diabetes with renal complications: OR = 1.07, 95% CI: 1–1.13, ^*^ *P* = 3.45 × 10^−2^. Type 1 diabetes with other specified/multiple/unspecified complications: OR = 1.12, 95% CI: 1.02–1.23, ^*^ *P* = 1.41 × 10^−2^. In addition, MR-Egger’ analysis portrayed that type 1 diabetes with ophthalmic complications: OR = 1.3, 95% CI: 1.04–1.62, ^*^ *P* = 4.43 × 10^−2^. Type 1 diabetes with neurological complications: OR = 1.19, 95% CI: 1.04–1.36, ^*^ *P* = 2.68 × 10^−2^. Weighted median analysis shows that type 1 diabetes (exclude DM2): OR = 1.14, 95% CI: 1.05–1.24, ^**^ *P* = 1.86 × 10^−3^. Type 1 diabetes with neurological complications: OR = 1.18, 95% CI: 1.08–1.28, ^***^ *P* = 1.48 × 10^−4^. Type 1 diabetes with other specified/multiple/unspecified complications: OR = 1.1, 95% CI: 1.01–1.2, ^*^ *P* = 2.37 × 10^−2^. Weighted mode analysis shows that type 1 diabetes (exclude DM2): OR = 1.15, 95% CI: 1.06–1.25, ^*^ *P* = 1.03 × 10^−2^. Type 1 diabetes with neurological complications: OR = 1.17, 95% CI: 1.07–1.28, ^**^ *P* = 5.07 × 10^−3^. Simple median analysis showed that type 1 diabetes with neurological complications: OR = 1.15, 95% CI: 1.03–1.28, ^*^ *P* = 1.26 × 10^−2^. The scatter plot (**Figure 2**) demonstrated that the five lines of “inverse variance weighted,” “MR-Egger,” “weighed median,” “weighed mode,” and “simple mode” all had consistent slope beta values and P values. Taking these findings together, we concluded that type 1 diabetes and complications had significant differences in liver fibrosis and cirrhosis and that they all satisfied the positive relationship, i.e., the higher the risk of having type 1 diabetes and complications. Cochran’s IVWQ test results revealed that there was no discernible heterogeneity in these IVs (**Table 1**). According to the outcomes of the MR-Egger regression intercept analysis, there was also no discernible directional horizontal pleiotropy (**Table 1**). However additional MR-PRESSO analysis likewise failed to detect any appreciable horizontal pleiotropy (**Table 1**).

**Table 1:** Trilinear table of type 1 diabetes and type 1 diabetes combined with acute and chronic complications and liver fibrosis and sclerosis levels of pleiotropy and heterogeneity analysis.

	Inverse variance weighted		MR-Egger		MR PRESSO
	Q	Q P	intercept	P	P
Type 1 diabetes	9.604	0.212	−0.039	0.466	0.321
Type 1 diabetes without complications	15.726	0.204	0.011	0.75	0.278
Type 1 diabetes with coma	10.449	0.107	0.054	0.472	0.248
Type 1 diabetes with ketoacidosis	10.65	0.831	0.011	0.639	0.843
Type 1 diabetes with neurological complications	10.507	0.572	−0.006	0.878	0.687
Type 1 diabetes with ophthalmic complications	17.83	0.164	0.014	0.725	0.189
Type 1 diabetes with renal complications	13.207	0.51	−0.01	0.714	0.597
Type 1 diabetes with other specified/multiple/unspecified complications	17.203	0.102	-0.007	0.871	0.209

## 4 Discussion

To determine the causal link between type 1 diabetes, complications, and liver fibrosis and cirrhosis, we conducted the first thorough bivariate multivariate MR analysis of type 1 diabetes, its acute and chronic consequences (exposure), and liver fibrosis and cirrhosis (outcome). According to our research, there is a causal association between type 1 diabetes, its acute and chronic consequences, and liver fibrosis and cirrhosis, implying that these conditions raise the likelihood of liver fibrosis advancement.

As a result, we surmised that T1D might hasten the development of liver fibrosis. Millions of people throughout the world suffer from type 1 diabetes (30), an inflammatory illness, and one of its complications is liver alterations (18, 31). We are aware that the long-term exposure of hepatocytes to glucose levels above the physiological range is a factor in the development of fibrosis (32), looking back at earlier investigations into type 1 diabetes and liver fibrosis. Patients with advanced fibrosis are more likely to suffer from metabolic syndrome, particularly if they have unfavorable lipid profiles, or are less sensitive to insulin compared with people without progressive fibrosis (15). Recent research reveals that sedentary lifestyles and high-calorie consumption are contributing factors in a rising proportion of T1D patients becoming overweight and developing insulin resistance (IR) features (33). Adipose tissue that is metabolically harmful is produced by insulin resistance in the liver and the periphery, which is increasingly thought to be associated with T1D (34). Adipose tissue inflammation is one of the factors contributing to steatohepatitis and fibrosis, especially in NAFLD (35), when type 1 diabetes is more severe or combined with complications manifested by elevated liver enzymes, which are typically linked to increased liver fat (6, 36). A similar relevance to glycogen deposition in hepatocytes has also been demonstrated in other studies (37). Hyperglycemia or insulin resistance, increased glycogen synthesis, stimulation of immunological cells, fibrogenic-programmed cells in the vasculature, or organ-specific parenchymal cells are all effects of inadequately managed T1D hyperglycemia (17). Therefore, we hypothesize that variations in insulinemia and hyperglycemia could possess a significant role in T1D-related liver fibrosis.

In addition, this study also discovered that patients with type 1 diabetes who experienced neurological and ophthalmic complications possessed ORs that were higher than the mean OR value for people with type 1 diabetes, whereas patients who experienced ketoacidosis and coma, renal complications, and other specified/multiple/unspecified complications experienced ORs that were lower than the mean OR value for people with type 1 diabetes, which is more in line with observational studies. The first observational study discovered an association between ALT and GGT activity and type 1 diabetes along with joint restriction and neuropathy, and GGT activity was also discovered to be associated with the severity of retinopathy (38). Another Dutch cross-sectional study revealed that concomitant neuropathy and ophthalmopathy were considerably more prevalent in the 150 patients with type 1 diabetes than coexisting renal and cardiovascular illness, but unfortunately, the number of participants was insufficient to draw any conclusions. Unfortunately, a correlation factor analysis (15) was not possible due to the small number of patients with type 1 diabetes and its comorbidities. Recent studies (39, 40) have additionally demonstrated that people with severe insulin-deficient diabetes are more likely to develop retinopathy and neuropathy. In this context, we hypothesize that type 1 diabetes with neurological and ocular problems is more likely to encourage the development of liver inflammation and fibrosis in comparison with type 1 diabetes with other comorbidities. Meanwhile, the OR of type 1 diabetes with chronic complications was slightly greater than the OR of type 1 diabetes without complications and acute complications. However, despite the fact that the data were high and the ORs and 95% CI of type 1 diabetes patients without complications could not significantly differ from those of patients with combined complications, it is possible that this is because the patients included in the study did not have enough combined multisystem complications to be separated, the complications were not identified, or the lesions were mild but not to the point where other complications could be diagnosed, etc., or the difference between the progression of liver fibrosis solely and the presence or absence of combination problems in type 1 diabetes was not significant.

Unquestionably, this study discovered that type 1 diabetes and its consequences, to a greater or lesser extent, speed up the progression of liver fibrosis, which has ramifications for both current and future generations’ clinical and public health. In this regard, our recommendation is to make an effort to manage blood glucose in type 1 diabetes patients in order to lessen the progression of multiple diseases such as liver fibrosis and to slow down oscillations in glucose and insulinemia. Future research is required in order to better comprehend the mechanisms behind the association between type 1 diabetes, its concomitant conditions, and the risk of cirrhosis and liver fibrosis.

## 5 Strengths and limitations

### 5.1 Strengths

We conducted the first Mendelian randomization study to effectively examine the causal association between type 1 diabetes and its acute and chronic consequences, including liver fibrosis and cirrhosis. The implementation of this strategy contains various benefits. First, we were able to lessen the potential effects of confounding variables and the reverse causality demonstrated in observational research by employing randomly assigned variations as instrumental variables. Secondly, to increase the accuracy of the estimations, we included data from studies with larger sample sizes (>18,000 for type 1 diabetes and both its acute and chronic sequelae, and up to 214,403 for liver fibrosis and cirrhosis). Following that, we combined data from more recent investigations with the FinnGen database in our analysis. Finally, a variety of techniques (MR-Egger, weighted median, MR-PRESSO, weighted mode, simple median, and elimination of outlier pleiotropic SNPs) were employed to identify and compensate for any pleiotropy bias.

### 5.2 Limitations

First off, as genetic data were lacking, we were only able to do analyses on Europeans for type 1 diabetes, its acute and chronic consequences, and liver fibrosis and sclerosis. Genetic variety makes it challenging to make firm conclusions about the causes of racial differences, so our findings might not be generalizable to other racial populations. However, we have no knowledge of any information indicating that the majority of ethnic groups should not be included in the current study. Secondly, it was impossible to separate the enormous number of patients with concomitant multisystem illnesses who were included in the study, and we did not have enough confidence that each complication was a different illness; thus, the ORs did not significantly differ across groups.

## 6 Conclusions

According to our MR investigation, type 1 diabetes and both its acute and chronic consequences might increase the likelihood that liver fibrosis could develop. The development of liver inflammation and fibrosis is more likely to be promoted by type 1 diabetes with neurological and ocular problems, which offers fresh insights into the genetic and clinical research of liver fibrosis and cirrhosis. Future research is required to clarify the probable processes underlying how type 1 diabetes, as well as its acute and chronic consequences, contribute to liver fibrosis.

## Data availability statement

The original contributions presented in the study are included in the article/**Supplementary Material**. Further inquiries can be directed to the corresponding author.

## Ethics statement

This research has been conducted using published studies and consortia providing publicly available summary statistics. All original studies have been approved by the corresponding ethical review board, and the participants have provided informed consent. In addition, no individual-level data were utilized in this study. Therefore, no new ethical review board approval was required. The studies were conducted in accordance with the local legislation and institutional requirements. Written informed consent for participation in this study was provided by the participants’ legal guardians/next of kin. Written informed consent was obtained from the minor(s)’ legal guardian/next of kin for the publication of any potentially identifiable images or data included in this article.

## Author contributions

GH: Writing – original draft, Visualization, Validation, Software, Resources, Methodology, Investigation, Formal analysis, Data curation, Conceptualization. YG: Writing – review & editing, Supervision, Project administration, Funding acquisition.
