# Supplementary material for: Type 1 diabetes and combined acute and chronic complications are associated with risk of progression of liver fibrosis: a Mendelian randomization study
Source: Front Endocrinol (Lausanne). 2024 Aug 5;15:1302611. doi: 10.3389/fendo.2024.1302611 (PMC11330757; doi:10.3389/fendo.2024.1302611)
Supplement: Supplementary file 1 [file DataSheet_1.pdf]

	Inverse variance weighted		MR-Egger		MR PRESSO
	Q	Q P	intercept	P	P
Type 1 diabetes	9.604	0.212	-0.039	0.466	0.321
Type 1 diabetes without complications	15.726	0.204	0.011	0.75	0.278
Type 1 diabetes with coma	10.449	0.107	0.054	0.472	0.248
Type 1 diabetes with ketoacidosis	10.65	0.831	0.011	0.639	0.843
Type 1 diabetes with neurological complications	10.507	0.572	-0.006	0.878	0.687
Type 1 diabetes with ophthalmic complications	17.83	0.164	0.014	0.725	0.189
Type 1 diabetes with renal complications	13.207	0.51	-0.01	0.714	0.597
Type 1 diabetes with other specified/multiple/unspecified complications	17.203	0.102	-0.007	0.871	0.209
