# Supplementary material for: Type 1 diabetes and combined acute and chronic complications are associated with risk of progression of liver fibrosis: a Mendelian randomization study
Source: Front Endocrinol (Lausanne). 2024 Aug 5;15:1302611. doi: 10.3389/fendo.2024.1302611 (PMC11330757; doi:10.3389/fendo.2024.1302611)
Supplement: Supplementary file 2 [file DataSheet_2.pdf]

Type 1 diabetes

SNP	chr	Pos	EA/OA	SNP-exposure			SNP-outcome		
				beta	SE	*p*	beta	SE	*p*
rs10947458	6	33816451	T/C	-0.252	0.035	9.42E-13	0.033	0.056	0.561
rs1233386	6	29558190	T/C	0.451	0.042	6.74E-27	0.026	0.07	0.711
rs1794269	6	32673894	T/C	1.177	0.031	1.00E-200	0.195	0.053	0.0002
rs689	11	2182224	T/A	0.579	0.041	2.58E-45	0.008	0.062	0.895
rs707958	6	33048599	A/C	-0.282	0.031	2.24E-19	0.006	0.052	0.908
rs9260231	6	29914866	T/G	-0.577	0.065	6.65E-19	0.073	0.097	0.454
rs9264277	6	31224667	C/T	0.303	0.035	4.39E-18	0.142	0.057	0.014
rs9468618	6	29750776	T/C	-0.448	0.072	4.28E-10	-0.094	0.11	0.392

Type 1 diabetes without complications

SNP	chr	Pos	EA/OA	SNP-exposure			SNP-outcome		
				beta	SE	*p*	beta	SE	*p*
rs11571297	2	204745003	C/T	-0.136	0.024	1.16E-08	-0.104	-0.104	0.046
rs11637189З	6	26359866	A/G	-0.242	0.035	4.27E-12	0.039	0.039	0.595
rs193474	22	30449094	G/A	-0.141	0.025	1.00E-08	-0.009	-0.009	0.861
rs2023497	6	29551333	C/T	0.297	0.025	3.97E-33	0.11	0.11	0.04
rs55895438	6	30583723	C/T	0.69	0.068	2.37E-24	-0.019	-0.019	0.897
rs689	11	2182224	T/A	0.495	0.031	1.79E-58	0.008	0.008	0.895
rs705702	12	56390636	G/A	0.168	0.025	1.90E-11	-0.067	-0.067	0.222
rs7090530	10	6110875	A/C	0.14	0.025	1.50E-08	-0.032	-0.032	0.556
rs74203920	21	45714294	T/C	0.353	0.06	4.52E-09	0.077	0.077	0.559
rs9264277	6	31224667	C/T	0.243	0.027	4.30E-20	0.142	0.142	0.014
rs9272324	6	32604152	G/A	0.917	0.025	1.00E-200	0.136	0.136	0.012
rs9275855	6	32691266	C/T	-0.662	0.028	3.49E-128	-0.011	-0.011	0.857
rs9468618	6	29750776	T/C	-0.398	0.054	1.95E-13	-0.094	-0.094	0.392

Type 1 diabetes with coma

SNP	chr	Pos	EA/OA	SNP-exposure			SNP-outcome		
				beta	SE	*p*	beta	SE	*p*
rs11538043C	6	29849257	C/A	0.904	0.142	1.67E-10	0.137	0.17	0.42
rs375555	6	33557741	C/T	0.53	0.058	4.13E-20	-0.021	0.069	0.759
rs3957146	6	32681530	C/T	1.915	0.071	5.70E-159	0.096	0.078	0.22
rs62404042	6	32404276	A/T	-0.736	0.093	2.78E-15	-0.277	0.107	0.01
rs6679677	1	114303808	A/C	0.518	0.06	9.50E-18	0.188	0.071	0.008
rs689	11	2182224	T/A	0.543	0.056	2.42E-22	0.008	0.062	0.895
rs9265832	6	31311480	A/G	0.491	0.044	2.20E-29	0.056	0.052	0.28

Type 1 diabetes with ketoacidosis

SNP	chr	Pos	EA/OA	SNP-exposure			SNP-outcome		
				beta	SE	*p*	beta	SE	*p*
rs11138873	9	83469092	C/T	0.325	0.071	4.94E-06	-0.04	-0.04	0.638
rs14932485Ƽ	14	19483931	A/C	3.654	0.797	4.60E-06	0.104	0.104	0.897
rs1683253	1	114638577	C/T	0.193	0.042	3.40E-06	0.052	0.052	0.299
rs17321459	8	123331040	T/A	0.226	0.047	1.43E-06	-0.015	-0.015	0.785
rs231764	2	204723541	T/C	0.19	0.042	4.60E-06	0.068	0.068	0.174
rs2476601	1	114377568	G/A	-0.623	0.059	7.63E-26	-0.179	-0.179	0.011
rs2855433	6	33158018	T/G	-0.213	0.044	1.32E-06	-0.002	-0.002	0.964
rs2905763	6	29913806	A/C	-0.551	0.07	3.72E-15	-0.006	-0.006	0.938
rs3957146	6	32681530	C/T	2.054	0.068	1.00E-200	0.096	0.096	0.22
rs41159	22	30407364	A/G	-0.207	0.043	1.66E-06	-0.031	-0.031	0.552
rs45619336	21	45814051	T/C	0.576	0.117	7.82E-07	-0.035	-0.035	0.806
rs61938963	12	56446804	T/C	0.211	0.044	1.77E-06	-0.051	-0.051	0.341
rs689	11	2182224	T/A	0.614	0.056	2.73E-28	0.008	0.008	0.895
rs7751923	6	31360257	T/G	0.325	0.054	2.07E-09	0.091	0.091	0.167
rs7988875	13	41517508	A/C	0.348	0.075	3.74E-06	0.035	0.035	0.698
rs915894	6	32190390	G/T	0.857	0.047	1.35E-75	0.083	0.083	0.145
rs9380121	6	29337817	A/G	0.3	0.059	4.46E-07	-0.012	-0.012	0.867

Type 1 diabetes with neurological complications

SNP	chr	Pos	EA/OA	SNP-exposure			SNP-outcome		
				beta	SE	*p*	beta	SE	*p*
rs10889932	1	72229193	A/G	-0.294	0.06	9.12E-07	-0.128	0.051	0.012
rs1794269	6	32673894	T/C	1.153	0.063	9.46E-75	0.195	0.053	0.0002
rs2723840	12	11953903	G/C	0.281	0.06	3.03E-06	0.027	0.051	0.6
rs2765329	13	101606631	C/T	-0.754	0.149	4.02E-07	-0.062	0.118	0.599
rs34184819	6	150653219	G/A	0.549	0.112	8.59E-07	0.144	0.092	0.116
rs55940417	14	101177015	A/C	0.946	0.204	3.63E-06	0.176	0.159	0.267
rs62236610	22	27989342	G/A	0.443	0.095	3.30E-06	-0.007	0.08	0.927
rs6679677	1	114303808	A/C	0.534	0.086	6.03E-10	0.188	0.071	0.008
rs689	11	2182224	T/A	0.438	0.076	7.88E-09	0.008	0.062	0.895
rs707958	6	33048599	A/C	-0.331	0.061	5.23E-08	0.006	0.052	0.908
rs75165919	6	27872898	A/G	0.549	0.111	6.69E-07	0.194	0.095	0.04
rs75428932	11	102882015	T/A	0.605	0.131	4.08E-06	0.084	0.106	0.426
rs76458667	17	76144468	T/C	0.541	0.106	3.62E-07	0.022	0.088	0.805

Type 1 diabetes with ophthalmic complications

SNP	chr	Pos	EA/OA	SNP-exposure			SNP-outcome		
				beta	SE	*p*	beta	SE	*p*
rs115380430	6	29849257	C/A	0.837	0.094	3.71E-19	0.137	0.17	0.42
rs12525616	6	30900435	T/G	0.603	0.066	7.15E-20	0.068	0.115	0.554
rs375555	6	33557741	C/T	0.488	0.039	2.10E-35	-0.021	0.069	0.759
rs62404591	6	32202920	C/G	-0.84	0.071	4.08E-32	-0.304	0.114	0.008
rs6679677	1	114303808	A/C	0.554	0.042	1.53E-40	0.188	0.071	0.008
rs689	11	2182224	T/A	0.541	0.039	8.41E-44	0.008	0.062	0.895
rs705700	12	56389293	C/T	0.184	0.03	8.88E-10	-0.067	0.052	0.193
rs7234029	18	12877060	G/A	0.218	0.038	1.49E-08	0.009	0.067	0.89
rs74203920	21	45714294	T/C	0.497	0.077	1.21E-10	0.077	0.132	0.559
rs9264277	6	31224667	C/T	0.305	0.034	8.82E-20	0.142	0.057	0.014
rs9265832	6	31311480	A/G	0.547	0.031	5.34E-72	0.056	0.052	0.28
rs9296104	6	33917860	G/A	-0.175	0.03	3.10E-09	-0.011	0.051	0.828
rs9468618	6	29750776	T/C	-0.378	0.067	2.17E-08	-0.094	0.11	0.392

Type 1 diabetes with renal complications

SNP	chr	Pos	EA/OA	SNP-exposure			SNP-outcome		
				beta	SE	*p*	beta	SE	*p*
rs114379727	6	30316917	A/G	1.018	0.156	6.34E-11	0.036	0.161	0.823
rs142400090	8	54015533	G/A	0.722	0.155	3.10E-06	-0.184	0.158	0.245
rs17482943	10	78942437	A/G	0.236	0.051	4.57E-06	0.057	0.055	0.298
rs182084602	17	70250916	C/T	0.738	0.159	3.63E-06	0.099	0.164	0.547
rs212079	16	16220126	T/C	0.446	0.092	1.35E-06	0.137	0.098	0.162
rs2248151	6	29914548	T/C	-0.431	0.076	1.36E-08	-0.012	0.079	0.875
rs2476601	1	114377568	G/A	-0.546	0.068	6.16E-16	-0.179	0.07	0.011
rs3957146	6	32681530	C/T	1.917	0.082	1.24E-119	0.096	0.078	0.22
rs41174	22	30426069	T/C	-0.245	0.051	1.30E-06	0.009	0.054	0.869
rs5744494	5	66485816	T/A	0.599	0.13	4.09E-06	-0.022	0.135	0.868
rs689	11	2182224	T/A	0.349	0.06	6.30E-09	0.008	0.062	0.895
rs705699	12	56384804	A/G	0.244	0.048	4.52E-07	-0.066	0.052	0.2
rs7172126	15	96025293	C/A	-0.228	0.049	2.91E-06	0.045	0.052	0.386
rs7867327	9	15082530	A/G	-0.267	0.056	1.94E-06	-0.029	0.059	0.628
rs915894	6	32190390	G/T	0.709	0.053	3.42E-40	0.083	0.057	0.145

Type 1 diabetes with other specified/multiple/unspecified complications

SNP	chr	Pos	EA/OA	SNP-exposure			SNP-outcome		
				beta	SE	*p*	beta	SE	*p*
rs10876864	12	56401085	A/G	-0.152	0.027	1.87E-08	0.045	0.051	0.373
rs1611236	6	29748690	A/G	-0.199	0.033	1.61E-09	0.125	0.063	0.047
rs2745400	6	29488249	G/A	-0.171	0.028	7.95E-10	-0.076	0.053	0.15
rs6679677	1	114303808	A/C	0.514	0.038	4.04E-41	0.188	0.071	0.008
rs689	11	2182224	T/A	0.477	0.035	2.10E-41	0.008	0.062	0.895
rs74203920	21	45714294	T/C	0.387	0.07	3.61E-08	0.077	0.132	0.559
rs7764080	6	33743741	T/C	0.464	0.04	6.82E-32	0.031	0.071	0.664
rs77647805	6	33119682	A/G	-0.296	0.052	1.31E-08	0.019	0.095	0.843

rs9264277	6	31224667	C/T	0.251	0.031	2.12E-16	0.142	0.057	0.014
rs9265753	6	31303939	C/G	0.637	0.038	4.89E-63	0.026	0.067	0.697
rs9274663	6	32636521	A/G	1.52	0.041	1.00E-200	0.169	0.075	0.024
rs9468618	6	29750776	T/C	-0.339	0.061	3.13E-08	-0.094	0.11	0.392
